# mintRULS: Prediction of miRNA–mRNA Target Site Interactions Using Regularized Least Square Method

**DOI:** 10.3390/genes13091528

**Published:** 2022-08-25

**Authors:** Sushil Shakyawar, Siddesh Southekal, Chittibabu Guda

**Affiliations:** 1 Department of Genetics, Cell Biology and Anatomy, University of Nebraska Medical Center, Omaha, NE 68198, USA; 2 Center for Biomedical Informatics Research and Innovation (CBIRI), University of Nebraska Medical Center, Omaha, NE 68198, USA

**Keywords:** miRNA–target site interaction, least square regression, nucleotide sequence feature, pairwise feature scoring

## Abstract

Identification of miRNA–mRNA interactions is critical to understand the new paradigms in gene regulation. Existing methods show suboptimal performance owing to inappropriate feature selection and limited integration of intuitive biological features of both miRNAs and mRNAs. The present regularized least square-based method, mintRULS, employs features of miRNAs and their target sites using pairwise similarity metrics based on free energy, sequence and repeat identities, and target site accessibility to predict miRNA-target site interactions. We hypothesized that miRNAs sharing similar structural and functional features are more likely to target the same mRNA, and conversely, mRNAs with similar features can be targeted by the same miRNA. Our prediction model achieved an impressive AUC of 0.93 and 0.92 in LOOCV and LmiTOCV settings, respectively. In comparison, other popular tools such as miRDB, TargetScan, MBSTAR, RPmirDIP, and STarMir scored AUCs at 0.73, 0.77, 0.55, 0.84, and 0.67, respectively, in LOOCV setting. Similarly, mintRULS outperformed other methods using metrics such as accuracy, sensitivity, specificity, and MCC. Our method also demonstrated high accuracy when validated against experimentally derived data from condition- and cell-specific studies and expression studies of miRNAs and target genes, both in human and mouse.

## 1. Introduction

The process of microRNA (miRNA)-directed silencing of messenger RNA (mRNA) has been described as another layer of gene regulatory mechanism in many organisms including animals and plants. By means of regulating gene expression at the post-transcriptional level, miRNA are involved in a wide range of biological processes such as cell development and maintenance [1], cell-to-cell interactions [2], and cancer growth and progression [3]. Around 90% of human genes are governed and regulated by one or more miRNAs at the post-transcriptional level [4].

Factually, single miRNA can interact with multiple mRNAs and individual mRNA can also be targeted by several miRNAs, forming a far more complex network of gene regulation [5,6], which is challenging to study and understand. The interaction between miRNA (average ~22-nt) and its target mRNA involve a seed region (~2–8 nucleotide long) on the miRNA, which seeks a complementary site mostly in the 3′ untranslated region (UTR) of mRNA to bind with; however, perfect seed pairing (canonical interaction) is not required to form a miRNA–mRNA complex in a so-called non-canonical interaction [7,8]. In previous studies, miRNA binding sites have also been identified in the 5′ UTR and coding regions [9,10]. These interactions have shown silencing effects on gene expression [11]. Recent studies also suggested that flanking regions (other than seed binding regions) at both ends of mRNA also contribute towards miRNA–mRNA interactions [12,13]. These studies reveal that the mechanisms involved in miRNA-based gene silencing are very complex and prediction of miRNA–mRNA interactions involves deploying multi-level characteristics of miRNA and their target sites.

Several bioinformatics-based approaches were developed to understand miRNA–mRNA interactions. These tools mainly adopted modulating features such as Watson–Crick pairings [14], the thermodynamic stability of miRNA and mRNA complexes [15], and binding site abundance, availability, and accessibility [15] to predict the interactions. Predictive methods such as TargetScan [16], miRWalk [17], MBSTAR [18], DeepMirTar [19], miRAW [20], and RPmirDIP [21] were developed to identify association between miRNAs and mRNAs. MBSTAR uses multiple instances of learning from validated miRNA binding sites to calculate interaction scores. miRDB database [22,23] includes a large collection of miRNA–mRNA interactions predicted by MirTarget tool (an inbuild component of miRDB), which was developed based on common features of miRNA binding sites extracted from high-throughput sequencing experiment. STarMir [24] adopts logistic modeling framework with crosslinking immunoprecipitation (CLIP) studies to predict miRNA binding sites. The model uses sequence-based features and targets secondary structures for predicting the binding sites. Recently, miRAW was developed to predict non-canonical interactions between miRNAs and target mRNAs [20]. Similarly, TargetScan used 14 different sequence features to predict miRNA–mRNA interactions. In continuation, various databases were developed based on these algorithms to provide predicted and experimentally verified miRNA–mRNA interaction pairs. The most common databases that provide predicted miRNA–mRNA interactions include miRDB, TarBase [25], and miRTarBase [26]. Previous reviews also described working strategies, data integration, feature extraction, and limitations of the existing methods [27,28,29].

Early prediction tools such as GUUGle [30] have utilized a single feature based on ‘seed base pairing’ for prediction. However, most methods as mentioned above eventually adopted multiple features that include seed pairing, free energy, sequence conservation, and target site accessibility that were derived from known miRNA–mRNA interaction pairs. These tools showed inconsistencies in their predictions because of inadequate emphasis given to the selection of context-specific features and their weights to reflect the characteristic environment for miRNA–target interactions. For example, algorithms focusing on the sequence conservation strategy work better only for phylogenetically closer species. One of such methods includes the miRanda algorithm [31], which considered the conservation of miRNA binding sites and positions in 3′ UTR to identify potential miRNA–target interactions only in closely related species. Furthermore, the strategies for extracting and integrating the structural and functional features shared between multiple miRNAs that could be responsible for targeting same mRNAs have been less emphasized in previous approaches [14,32]. In other words, similarity-based feature integration strategies have not been much explored in this context. However, a recent tool, miRTMC [33] was developed by adopting similarity networks of miRNAs and mRNAs, and miRNA–mRNA interaction networks. Apart from this, the datasets used to train and test these models are consistent, leading to small overlap between predicted targets by different methods, as highlighted in the previous articles and reviews [14,27,28,34]. Subsequently, most tools suffer from poor sensitivity and accuracy when comparisons are made against experimental data [29,35], raising the need for developing more sophisticated computational methods.

Here, we develop a new approach, called mintRULS (microRNA–Target Interaction Prediction Using Kronecker-Regularized Least Square classification), which incorporates sensitive features from miRNAs and target sites on mRNAs in a pairwise manner by utilizing least-square regression-based classification to predict interactions between them. We hypothesized that miRNAs with shared features are more likely to interact with the same mRNA, while mRNAs with similar features tend to be targeted by the same miRNA. With this hypothesis, our strategy of utilizing the similarity features within the miRNA and mRNA species has helped overcome the limitations of the current prediction methods. We demonstrate that our model outperforms the existing tools in the prediction accuracy and validate the method using experimental gene expression data from human and mouse, which will help improve our understanding of miRNA-associated gene regulation at the post-transcriptional level.

## 2. Materials and Methods

### 2.1. miRNA–Target Site Associations in Human and Mouse

A subset of the dataset from a previous study [36] was utilized in the present analysis. The data include miRNA and miRNA target site (miTS) associations (MTAs) from (i) study of miRNA interactome by CLASH (crosslinking, ligation, and sequencing of hybrids) in HEK293 cells [8] and (ii) miRNA-target site interaction data in MirTarBase 8.0 with experimental evidence (immunoblot, luciferase reporter assay, qRT-PCR). The combined data were preprocessed to remove pairs with incomplete information. For example, all miRNAs with one or more “N” letters in their nucleotide sequences were removed; whereas, any target sites with >50% “N” letters were filtered out from the study. The final human dataset contains 34,413 MTAs between 845 miRNAs and 32,709 miTS (from 17,625 human mRNA transcripts), while mouse dataset includes 2829 experimentally verified interactions between 327 miRNA and 2675 miTS (from 2424 mRNA transcripts: Unannotated: 1925, annotated genes: 499). For better description, the adjacency matrices $A_{845\times 32709}$ and $A_{327\times 2675}$ were generated for human and mouse datasets, respectively. The experimentally verified pairs in each matrix represent positive dataset, whereas the remaining pairs were considered as negative dataset.

### 2.2. Kernel Similarity Scores for miRNA

We developed a comprehensive scoring scheme by using relevant features that are more likely to discriminate between the binding and non-binding MTAs. The rationale for including each feature is provided below.

#### 2.2.1. Free Energy (FE)-Based Similarity

Free energy of RNA molecules (miRNAs and mRNAs) is a very important property that facilitates their interactions because the energy is involved in unfolding the interaction sites to allow pairing of nucleotides between miRNAs and mRNAs. Therefore, lower overall free energy means higher stability of the miRNA–mRNA complex, which can be interpreted as higher possibility of the real interactions. Long et al., 2007 also found a correlation between the folded structure of mRNA and efficacy of miRNAs-driven repression [37]. This concept has also been previously used for the development of various miRNA–mRNA interaction prediction tools such as MiRNATIP [38], Avishkar [39], RNAhybrid [40], and other algorithms [41]. In the current work, Python package, seqfold (https://pypi.org/project/seqfold/, accessed on 28 March 2022) was used to calculate the minimum free energy of each miRNA. This program takes the nucleotide sequence of a given miRNA as input to calculate free energy (also referred as folding energy) based on the thermodynamic principles. The FE-based pairwise similarity between two miRNAs $m_{i}$ and $m_{j}$ is calculated as Euclidean distance (Appendix A, Equation (A1)) and is denoted as $FE_{m}\left(m_{i},\;m_{j}\right)$. The pairwise matrix representing FE-based similarity between all miRNAs is denoted as $FE_{m}$.

#### 2.2.2. Gaussian Interaction Profile (GP) Kernel Similarity (Based on Known Associations)

The application of GP-based similarity has been successfully implemented in predicting drug–target interactions [42,43], drug–drug interactions [44], and miRNA–disease associations [45]. Here, GP kernel similarity between two miRNAs, $m_{i}$ and $m_{j}$, is defined as $GP_{m}\left(m_{i},\;m_{j}\right)$.

(1) $$GP_{m}\left(m_{i},\;m_{j}\right)=e^{\left(-\varphi_{m}\;\|\;\mathrm{IP}\left(m_{i}\right)-\;\mathrm{IP}\left(m_{j}\right){\|}^{2}\right)}$$

$\mathrm{IP}\left(m_{i}\right)$ is the binary vector representing the interaction profile of miRNA, $m_{i}$. $\varphi_{m}$ is selected to adjust the kernel width and can be calculated as:

(2) $$\varphi_{m}={\;\varphi }_{m}\prime /(\frac{1}{nm}\overset{nm}{\underset{i=1}{\sum }}\|\mathrm{IP}\left(m_{i}\right){\|}^{2})$$

*nm* equals the total number of selected miRNAs.

Based on previous studies [46], $\varphi_{m}\prime$ is set to 1. As defined above, pairwise matrix of GP-based similarities of selected miRNAs is denoted as $\;GP_{m}$.

#### 2.2.3. Needleman’s Sequence Similarity

As evident from experimentally verified miRNA-target pairs, miRNA with similar seed sequences are more likely to regulate a similar set of genes [47]. Based on this line of thought, the sequence-based pairwise similarity score was calculated using Needleman–Wunsch algorithms [48]. The similarity score between two miRNAs, $m_{i}$ and $m_{j}$ is denoted as $\;NS_{m}\left(m_{i},\;m_{j}\right)$, and the whole pairwise matrix is represented by $\;NS_{m}$.

#### 2.2.4. Simple Sequence Repeats (SSRs)-Based Similarity

SSRs are repetitive nucleotide sequences and are considered as important binding signatures embedded at the genetic level. Previous study found that miRNAs binding to complementary regions with SSRs showed perturbation in the RNA cross-talks in case of myotonic dystrophy type 1 (DM1) and type 2 (DM2) [49]. Considering the significance of SSRs in mRNA binding, we extracted repeat motifs (RF) from each miRNA using ssrtool (https://archive.gramene.org/db/markers/ssrtool, accessed on 20 November 2021). With the filtering criteria of minimum 3 repeats, we found 12 di-, 51 tri-, and 32 tetramers in all miRNAs. Considering the repeat counts in each miRNA, the Gaussian profile based pairwise similarity $SR_{m}\left(m_{i},\;m_{j}\right)$ between miRNAs, $m_{i}$ and $m_{j}$ are calculated as follows:

(3) $$SR_{m}\left(m_{i},\;m_{j}\right)=e^{\left(-\varphi_{m}\;\|\;\mathrm{RF}(m_{i})\;-\;\mathrm{RF}\left(m_{j}\right){\|}^{2}\right)}$$

where $\mathrm{RF}\left(m_{i}\right)$ and RF $\left(m_{j}\right)$ are binary vectors representing all RFs in miRNAs $m_{i}$ and $m_{j}$. Again, $\varphi_{m}$ is selected to adjust the kernel width and can be calculated as:

(4) $$\varphi_{m}=\varphi_{m}\prime /(\frac{1}{nm}\overset{nm}{\underset{i=1}{\sum }}\|\mathrm{RF}\left(m_{i}\right){\|}^{2})$$

As explained above, $\varphi_{m}\prime$ is set to 1 in this case. *nm* is the total number of selected miRNA and $SR_{m}$ represents the corresponding pairwise matrix of SR-based similarities.

#### 2.2.5. Integration of miRNA Similarity Scores

All four types of feature scores were combined by employing a weighted combination approach to obtain an integrated similarity matrix, $\;S_{m}$, as defined below:

(5) $$\;\;\;\;\;\;\;\;\;\;S_{m}=\{(\alpha_{1}\times FE_{m})+\left(\alpha_{2}\times GP_{m}\right)+\left(\alpha_{3}\times NS_{m}\right)+\left(\alpha_{4}\times SR_{m}\right)\}/\overset{4}{\underset{i=1}{\sum }}\alpha_{i}$$

where $\alpha_{i}$ represents weights given to the different similarities.

### 2.3. Kernel Similarity Scores for miTS

Similar to the scores for miRNAs, we employed a set of discriminatory features for miTS as follows.

#### 2.3.1. FE-Based Similarity between miTS

The *seqfold* tool was used in similar manner to calculate the minimum free energy of each miTS, followed by calculation of FE-based similarity between two miRNA binding sites, $t_{i}$ and $t_{j}$, as denoted by $\;FE_{t}\left(t_{i},\;t_{j}\right)$. The final symmetrical matrix of pairwise FE-based similarities is termed as, $FE_{t}$.

#### 2.3.2. Target Site Accessibility (TA)-Based Similarity

Accessibility of the miRNA target site is responsible for easing miRNA binding and subsequent miRNA-driven regulation [6,15]. We calculated accessibility of miTS using *RNAplfold* module of ViennaRNA package (http://www.tbi.univie.ac.at/RNA/, accessed on 20 November 2021). The pairwise similarity between TAs of two miTS $t_{i}$ and $t_{j}$ is calculated based on Euclidean distance and is denoted as $\;TA_{t}\left(t_{i},\;t_{j}\right)$. The matrix representing score for chosen miTS is termed as $\;TA_{t}$.

#### 2.3.3. AU Content (AU)-Based Similarity

mRNA can be folded to form a secondary structure which might hinder the repression potency of miRNA by lowering the site accessibility [50]. A previous study suggested that lowering the GC content (or high local AU content) near the target sites and also in the 3′ UTR region of mRNA increases accessibility to interact with miRNA [6,51]. Therefore, the GC content on each miTS was calculated separately, followed by calculation of pairwise AU-based similarity between two miTS, $t_{i}$ and $t_{j}$ based on Euclidean distance (Appendix A, Equation (A2)), and is dented by $AU_{t}\left(t_{i},\;t_{j}\right)$. The final similarity matrix of AU-based similarities between different miTS is represented by $AU_{t}$.

#### 2.3.4. Simple Sequence Repeats (SSRs)-Based Similarity

Similar to miRNAs, SSR motifs were extracted from each miTS with the same filtering criteria, and Gaussian profile-based pairwise similarity $SR_{t}\left(t_{i},\;t_{j}\right)$, between miTSs, $t_{i}$ and $t_{j}$ were calculated. Here, we denote the whole pairwise matrix of all miTS as $\;SR_{t}.$

#### 2.3.5. Integration of miTS’s Pairwise Similarities

Similar to the miRNAs analysis, different similarity matrices were combined with providing specific weightage $\beta_{i}$ to each one, as described below, to get final matrix $S_{t}$.

(6) $$\;S_{t}=\{(\beta_{1}\times FE_{t})+\left(\beta_{2}\times TA_{t}\right)+\left(\beta_{3}\times AU_{t}\right)+\left(\beta_{4}\times SR_{t}\right)\}/\overset{4}{\underset{i=1}{\sum }}\beta_{i}\;$$

$\beta_{i}$ provides weights given to a particular feature.

### 2.4. mintRULS

We developed a computational model, mintRULS, which utilizes known MTAs to predict possible interactions while incorporating multiple similarity-based kernels of miRNA and miTS. The relevance score is calculated based on Kronecker product and the regularized least square (RLS) method. The adjacency matrix, $A_{nm\times nt}$ was generated to describe the known and unknown associations between *nm* miRNAs and *nt* miTS. For known associations between miRNA $m_{i}$ and miTS $t_{j}$, the association value $A_{m_{i}\times t_{j}}$ was assigned 1, else 0.

**Figure 1:**
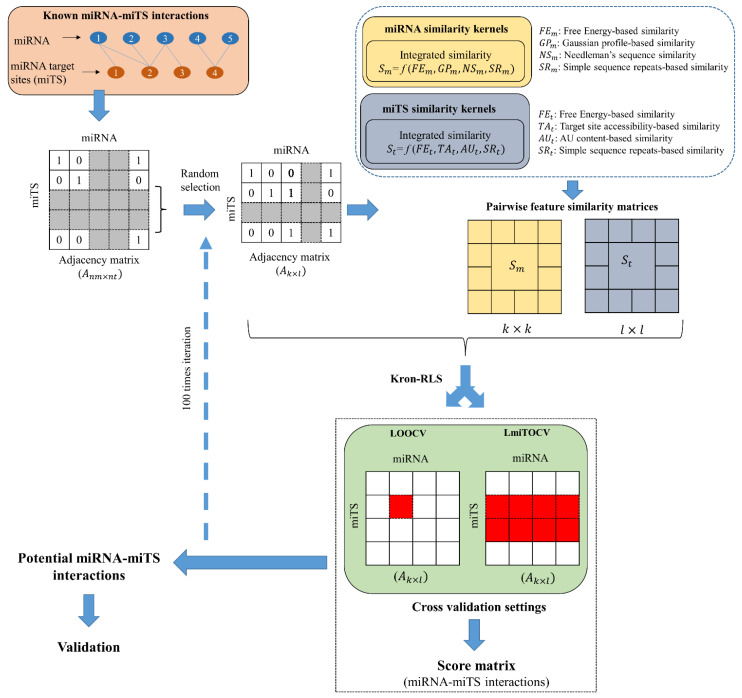
Schematic representation of the workflow for feature integration, cross-validation, and performance evaluation of the model mintRULS. miRNA: microRNA, miTS: miRNA Target Sites. CV: Cross-Validations, LOOCV: Leave-One-Out-CV (LOOCV), LmiTOCV: Leave-miTS-Out-CV. In the matrix $A_{nm\times nt}$, 1 represents positive interactions, while 0 represents no interactions between miRNA and target site.

As illustrated in Figure 1, out of the whole interaction data a random dataset with k number of miRNAs $M=\{m_{1},\;m_{2}$,…$m_{k}$}, and l number of target sites $T=\{t_{1},\;t_{2}$,…$t_{l}$} is selected to form random adjacency matrix $A_{k\times l}\subset A_{nm\times nt}$. The samples for training can be prepared as $S=\{(x_{1},\;y_{1}),\;(x_{2},\;y_{2}),\ldots (x_{n},\;y_{n})\}$, where $x_{i}\;\mathrm{and}\;y_{i}$ represent miRNA-miTS pair and corresponding binary level in the adjacency matrix, respectively with n=k×l.

Further, as explained in [52], using the labeled training samples S, the following objective function J is minimized with the goal of learning a function f to generalize it on new miRNA–miTS samples.

(7) $$J\left(f\right)=\overset{n}{\underset{i=1}{\sum }}{\left(y_{i}-f\left(x_{i}\right)\right)}^{2}+\lambda \|f{\|}_{K}^{2}$$

$\|f{\|}_{k}$ is the norm of function f measured in Hilbert space with kernel function K. The regularization parameter λ > 0 is adjusted for balancing prediction error and model complexity.

According to Representer Theorem [53], the function f in the above equation can be expressed in the following form to get minimizer of the objective function J.

(8) $$f\left(x_{i}\right)=\overset{n}{\underset{i=1}{\sum }}\alpha_{i}K\left(x,x_{i}\right)$$

As calculated in [54], ${\left|\left|f\right|\right|}_{K}^{2}=\alpha^{T}K\alpha$, the function can be represented as follows:

(9) $$minF\left(\alpha \right)=min\;\overset{n}{\underset{i=1}{\sum }}{\left(y-K\alpha \right)}^{T}\left(y-K\alpha \right)+\frac{\lambda }{2}\alpha^{T}K\alpha$$

As previously mentioned in [55], α in the above equation can be calculated by solving following linear equation:

(10) (K+λ×I)α=y

where K is the Kronecker product of two kernel similarities functions, K = $S_{m}⊗S_{t}$, with $S_{m}$ and $S_{t}$ as integrated similarity matrix of chosen miRNA and miTS. I is the identity matrix. As referred in the previous studies [56,57], the eigen decomposition of the kernel matrices $S_{m}$ and $S_{t}$ are performed as follows:

$$S_{m}=Q_{m}\Lambda_{m}Q_{m}^{T}\;\mathrm{and}\;S_{t}=Q_{t}\Lambda_{t}Q_{t}^{T}$$

In the above eigen decomposition, $Q_{m}$ and $Q_{m}^{T}$ represent eigenvalue vector and its transpose, respectively for miRNAs. Similar notations stand for miTS. $\Lambda_{m}$ and $\Lambda_{t}$ are the diagonal matrices. α in Equation (9) can be calculated as follows:

(11) $$\alpha =vec\left(Q_{m}CQ_{t}^{T}\right)$$

where

$$vec\left(C\right)=(\Lambda_{m}⊗\Lambda_{t\;})\left(\Lambda_{m}⊗\Lambda_{t}+\lambda \times I{)}^{-1}\right)vec\left(Q_{m}^{T}Y^{T}Q_{t}\right)$$

### 2.5. Cross-Validations and Performance Testing

#### 2.5.1. Cross-Validations

The performance of mintRULS model was evaluated by conducting cross-validation (CV) mainly in two ways: (1) Leave-One-Out-CV (LOOCV) and (2) Leave-miTS-Out-CV (LmiTOCV), using human and mouse datasets, separately. LOOCV refers to the condition when one MTA is considered as a test sample while the remaining ones in the adjacency matrix $A_{k\times l}$ are considered as training samples. In LmiTOCV, 10% of all miTS and their associations with miRNA are considered as test data while remaining MTAs in $A_{k\times l}$ are kept for training the model. To make the simulation process computationally inexpensive, the random k miRNA and l miTS are chosen from the original adjacency matrix $A_{nm\times nt}$ to form a sample adjacency matrix $A_{k\times l}$, with k=nm and l=0.1×nt. This randomization is iterated over 100 times to reduce impacts of data overfitting, and the model is simulated each time in both the environments, LOOCV and LmiTOCV.

#### 2.5.2. Score Normalization and Performance Evaluation

Actual and predicted miRNA-miTS interactions were used to calculate true positive rate (TPR), and false-positive rate (FPR). Receiver operating characteristics (ROC) curve was drawn to determine the area under ROC curve (AUC) for estimating the performance of the models. Additionally, other parameters such as accuracy, sensitivity, specificity, and MCC were also calculated for human and mouse datasets, separately. Minimum miTS sequence length as 40 and 30 nucleotides were considered to perform simulations in case of human and mouse, respectively. In the present analysis, AUC with values 0.5 meant the model can predict randomly, while AUC = 1 indicated the best performance of the model.

Further, mintRULS-predicted scores were normalized using unity-based methods to classify the miRNA-miTS pairs, as explained below:

(12) $$\;X^{\prime }=a+\frac{X-X_{min}}{X_{max}-X_{min}}\times \left(b-a\right)$$

where *a* = 0, and *b* = 1 was set in current model. $X^{\prime }$ is the derived normalized score of predicted score X for an interacting miRNA–miTS pair. $X_{min}\;\mathrm{and}\;X_{max}$ are minimum and maximum mintRULS score obtained for that miRNA across all miTS. The normalized score will provide space to define the strengths of the predicted interactions rather than classifying them in binary (on/off) relationships. All the pairs were divided into three categories based on quantile normalization of the score. The lower and upper quartile lines are considered as boundaries between each category, as defined below:

Weak Targets: <lower quartile (25th quartile).
Moderate Targets: between lower quartile (25th quartile) and upper quartile (75th quartile).
Strong Targets: >upper quartile (75th quartile).

#### 2.5.3. Comparison with Previous Methods

We also compared mintRULS predictions with the previous popular tools and databases which include miRDB, TargetScan, MBSTAR, RPmirDIP, and STarMir [24]. To make the comparison methodologically relevant and effective, we also included the tools whose working strategies directly or indirectly focus on features of miRNAs and their target sites. More specially, the objective here is to compare prediction power of mintRULS with other tools, which will subsequently help to understand importance of inclusion of multiple features (in pairwise manner) over single features. The interacting pairs predicted by these resources were obtained as of 20 March 2021.

MBSTAR is a machine learning program that extracts features from validated potential binding sites in the mRNA and use them to train the classifier and predict target and non-target mRNAs. Further, by using random forest classifier, the algorithm predicts functional binding sites in the mRNA. To choose a dataset of highly interacting miRNA–mRNA target pairs, all human sequence pairs with scores higher than 0.5 were considered as positive pairs and included in the present comparative analysis.

miRDB database contains miRNA-target pairs predicted by MirTarget, which is an algorithm trained by using crosslinking immunoprecipitation (CLIP)-based binding and miRNA expression data using the SVM machine learning framework. The algorithm looks for the common features which are associated with both miRNA and downregulation of the target. As a prediction score, the algorithm generates a probability score between 0 and 100 for each target site. In case of multiple target sites on mRNA, the individual score is combined to calculate final score. miRDB provides only interacting pairs with score > 50. Here, we downloaded all human interacting pairs and compared with mintRULS’s predictions.

STarMir, a web server, was developed on a logistic modeling framework and trained using CLIP data. The method incorporates a variety of thermodynamic, structural, and sequence-based features for seed and non-seed regions as well as different regions (e.g., (3′ UTR, CDS and 5′ UTR)) on mRNA. In terms of the prediction score, the model outputs the probability score representing miRNA–target site interactions. As discussed in the article, predictions with the probability score of 0.75 or higher give highly likely interacting pairs. Therefore, only highly interacting pairs were considered in this analysis for comparison.

TargetScan predicts miRNA–target interactions by matching conserved 8-mer, 7-mer, and 6-mer sites in the seed region. TargetScanHuman (v 7.2) (https://www.targetscan.org/vert_80/, accessed on 20 March 2021) utilizes various binding sites related characteristics and 14 features to predict interactions between miRNA and its targets. From the database, interacting pairs with weighted context++ score percentile higher than 50 were considered as positive pairs in the comparative analysis.

RPmirDIP provides interacting pairs predicted by mirDIP (microRNA Data Integration Portal) [58] which uses a semi-supervised machine learning method “Reciprocal Perspective (RP)”. In the present analysis, all the pairs with the recommended Difference of Scores (DoS) of higher than 0.5 were considered.

The separate data matrix representing interactions between miRNA and targets were prepared for each database discussed above. The interacting and non-interacting pairs in the test dataset were searched in each data matrix, and confusion matrix was built to calculate AUC values in each case.

### 2.6. Model Code Implementation and Software Availability

Python 3.7 (https://www.python.org), PyCharm Community version 2019.3 (https://www.jetbrains.com/pycharm/), and R 4.0.5 (https://www.r-project.org/) were used to develop scripts and run all the simulations, accessed on 20 November 2021. All the core scripts and related data can be accessed from https://doi.org/10.5281/zenodo.6360587.

### 2.7. Validation of Predictions

#### 2.7.1. Using Condition- and Cell-Specific Studies

Experimental data that identified interactions between hsa-miR-548ba and four genes (*IFR*, *PTEN*, *NEO1*, and *SP110*) in human ovarian granulosa cells [59] were used to validate the mintRULS predictions. Similarly, experimentally verified interactions of miRNA hsa-miR-34a-5p with genes including *JNK3*, *SMAD7*, *SMAD2*, *CREB1*, *TH*, *CLOCK*, *GRIA4*, and *PARK2* in Human Neuroblastoma Cell Line SH-SY5Y using high-throughput miRNA interaction reporter assay (HiTmIR) were also considered [60].

#### 2.7.2. Using Literature-Based Data

The top predictions by mintRULS were compared with the information in literature and databases including miRDB and TargetScan.

#### 2.7.3. Using Expression Data of miRNA and mRNA in Gastrointestinal (GI) Cancer

TCGA level 3 gene/mature miRNA expression data for pan-GI cancers (stomach adenocarcinoma, STAD; cholangiocarcinoma, CHOL; pancreatic adenocarcinoma, PAAD; esophageal carcinoma, ESCA; and liver hepatocellular carcinoma, LIHC) were collected and analyzed using QIAGEN Ingenuity Pathway Analysis (IPA) (please refer to Supplementary Document for the methodology of IPA) to identify negative expression correlations of top predicted miRNA–mRNA pairs from mintRULS.

#### 2.7.4. Using Expression Data of miRNA and mRNA in Normal and Septic Mice

The expression data of miRNAs (GSE74952 study) and genes (GSE55238 study) in control and septic mice, respectively, were downloaded from Gene Expression Omnibus (GEO) database and analyzed using GEO2R. The mintRULS predicted pairs that showed negative expression correlations were identified.

More methodological description of (c) and (d) are provided in Appendix A (method section).

## 3. Results

### 3.1. Performance Evaluation of mintRULS

**Figure 2:**
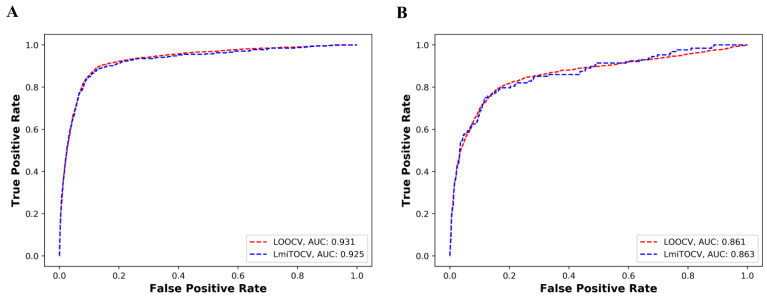
Performance of the mintRULS model using ROC profiling in case of (**A**) human, and (**B**) mouse datasets. miTS: mRNA target site, LOOCV: Leave-One-Out-Cross Validation, LmiTOCV: Leave-miTS-Out-Cross-Validation.

mintRULS achieved an average AUC of 0.93 and 0.92 on the human dataset, while it scored AUC of 0.861 and 0.865 on the mouse dataset in LOOCV and LmiTOCV simulation environments, respectively (Table 1). The ROC profile indicating AUC measurements in both the cases are shown in Figure 2A,B. The model also recorded high accuracy at 90.8% and 91% in LOOCV and LmiTOCV simulations, respectively, using human data, supporting its strong prediction ability. In the case of mouse also, the achieved accuracies were 84.6% and 84.4% in LOOCV and LmiTOCV settings (Table 1). For more intuitive evaluations, high measurements of the other parameters including MCC, specificity, and sensitivity (Table 1) indicated high performance of the model on human as well mouse datasets. In case of mouse, the prediction performance of the model has been observed to be comparatively similar in both the simulation environments. In addition, the high specificity indicates the better ability for identifying specific interactions between miRNA and miTS in the mouse. We therefore interpreted that the model has the ability to predict miRNA–target site interactions.

**Figure 3:**
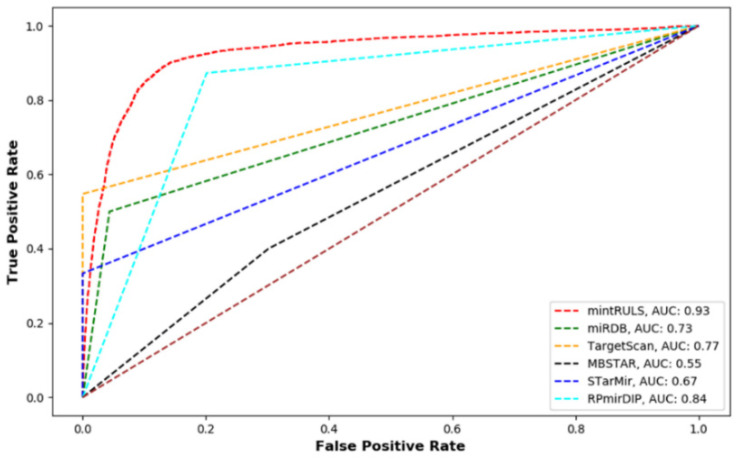
Performance comparisons between predictions made by mintRULS model and other previous methods that include miRDB, TargetScan, MBSTAR, RPmirDP, and STarMir, using Receiver operating characteristics (ROC) curve and Area Under Curve (AUC) determination. The dark red dashed diagonal line stands for a non-discriminatory test.

Further, comparison of mintRULS predictions with other methods were performed using the human dataset. The methods miRDB, TargetScan, MBSTAR, RPmirDIP, and STarMir achieved AUC of 0.73, 0.77, 0.55, 0.84, and 0.67, respectively; in comparison mintRULS received better AUC of 0.93, in LOOCV settings, showing superior performance of the current method (Figure 3).

### 3.2. Evaluation of Regularization Parameter (λ)

**Figure A1:**
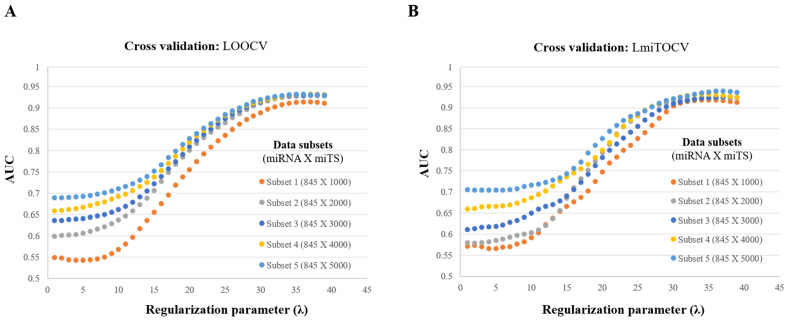
AUC measurement after simulating mintRULS model on different values of regularization parameter (λ). The randomization in case of each dataset was iterated over 100 times and average AUC was calculated in (**A**) LOOCV, and (**B**) LmiTOCV environment. Definitions: Subset 1 (845 X 100) contains a matrix representing interactions among 845 miRNAs and 1000 miTS; and similarly, for other subsets.

As defined in the method section, tuning the regularization parameter (λ) is important to reduce the overfitting which might decrease the variance of estimated regression parameters by adjusting the bias. Herein, we evaluated λ over different datasets in both LOOCV and LmiTOCV settings. Using the adjacency matrix $A_{nm\times nt}$, five different random data matrices, i.e., $A_{845\times 1000}$, $A_{845\times 2000}$, $A_{845\times 3000}$, $A_{845\times 4000}$, and $A_{845\times 5000}$ comprise of all 845 miRNAs and different numbers of random miTS, as shown in the subscript, were prepared. Figure A1 (Appendix A), indicated that a higher miTS number tends to provide better AUC in both LOOCV and LmiTOCV. However, it is not advisable to choose a larger number of miTS as it creates a very high number of empty cells in the adjacency matrix which eventually could lead to the underperformance of the model. Based on these results, we selected the dataset $A_{845\times 3000}$ as optimal for further analyses.

**Figure 4:**
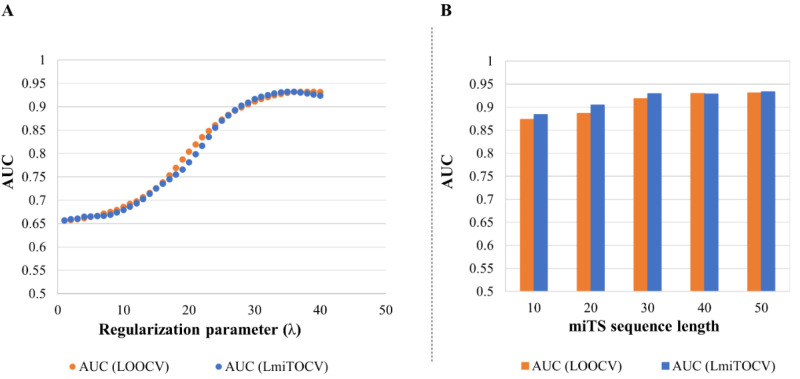
(**A**) Performance evaluation of regularization parameter (λ) in LOOCV and LmiTOCV simulation environments. The 100 times iterations of the data matrix A_(845 × 3000) (miRNA: 845 and miTS: 3000) was done with performing the model simulation. (**B**) Effect of variation on length of miTS sequences on the prediction performance of the model. As in the case of (**A**), randomized data matrix A_(845 × 3000) was used to perform the cross-validations in LOOCV and LmiTOCV environments. LOOCV: Leave-One-Out-Cross Validation; LmiTOCV: Leave-miTS-Out-Cross Validation; miRNA: MicroRNA; miTS: miRNA Target Site; AUC: Area Under the Receiver Operating Characteristic Curve.

Next, using the data matrix $A_{845\times 3000},$ AUC was measured for different values of regularization parameter λ. Interestingly, as shown in Figure 4A, λ > 35 obtained the highest values of AUC corresponding to 0.931 and 0.925 in the case of LOOCV and LmiTOCV, respectively, which we interpreted as optimal in our case. With the chosen λ = 35, the model extracts favorable features from miRNA and miTS sequence with adding some obvious biases to predict miRNA-miTS interactions.

### 3.3. Evaluation of miTS Sequence Length and Features

#### 3.3.1. Effect of Longer Sequence Length

The computational models have fully or partially utilized features associated with miTS sequences to predict interactions with miRNAs. As introduced earlier, GC content, accessibility, seed pairing, and flanking sequences are some of the widely used features in these models [15]; however, lack of emphasis has been given on consideration of the length of binding sites in most of the models. This is important mainly in the sense that an optimized length of miTS (including seed regions and flanking regions on both sides) can provide the best and effective features to predict more accurate interactions with miRNAs.

On this note, we performed systematic comparisons between different sequence lengths (=10, 20, 30, 40, and 50 nucleotides) of miTS to observe its impact on the model’s performance. As shown in Figure 4B, the higher sequence length corresponds to better AUC, suggesting more powerful and effective features. The shorter length of miTS may possibly cause high noises in the simulation, as also stated in [61]. However, for obvious reasons, too lengthy sequences might side pass any mutational effect on miTS, and are thus not recommended. Therefore, a sequence length of 40 nucleotides was considered as the most optimal in the current analysis.

#### 3.3.2. Feature Selection and Feature Contribution

**Figure 5:**
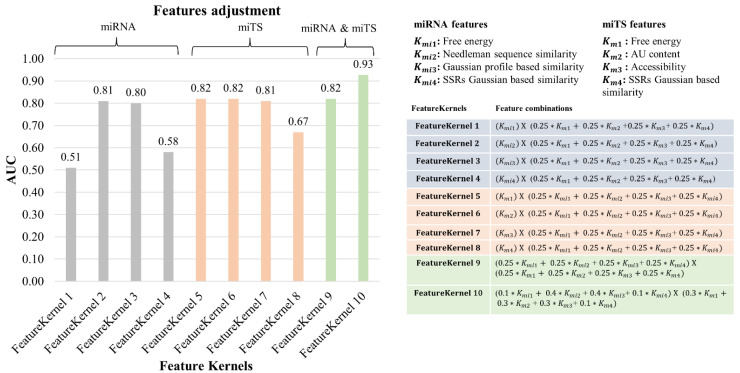
The model performance using different weights combinations of miRNA and mRNA target site features. SSR: Simple sequence repeats, miRNA: microRNA, miTS: miRNA Target Sites.

The model is generalized over different weight combinations used for prioritizing features of miRNA and miTS, separately. In this simulation process, the weights associated with mRNA features were kept constantly distributed to determine individual effect by miRNA’s features on model performance, as shown in Figure 5. In this case, Needleman sequence similarity and GP-based similarity showed higher contributions towards better performance of the model. Similarly, the effect of mRNA features was observed individually with no significant differences in the measured AUC values (Figure 5). Considering these findings, we simulated feature formulations giving more weightage to the features with more individual contributions and achieved significant improvements in AUC up to 0.93 (Figure 5). The model achieved higher AUCs of 0.81 and 0.80 for miRNA’s features, Needleman Sequence ($K_{mi2}$)-, and Gaussian profile ($K_{mi3}$)-based similarities, respectively, as compared to the other two features, free energy ($K_{mi1}$) and SSRs Gaussian-based similarity ($K_{mi4}$). The GP-based calculations, as their intrinsic characteristic, are done with the assumption that similar miRNAs can interact with the same targets, and vice versa, which is the base hypothesis of this study. It can also cover nonlinear relationship of known miRNA–target interactions. Previous successful applications of GP kernels include development of feature-based models for predicting drug–target interactions, miRNA–disease associations, circRNA–disease association, drug–disease associations, and drug–drug interactions [42,43,44,45,62]. Likewise, we also interpret that similarity-based models, including the current mintRULS, have the potential to predict miRNA–target interactions. On the other hand, SSR-based features, both from miRNA or mRNA, were not so predictive, perhaps because of the non-specificity of SSRs (i.e., *n* = 3 or 4 or 5) considered in the present study. As there are a handful of studies showing significance of SSRs in miRNA-target binding [49,63,64], further investigation on feature manipulation is required to better incorporate these features in the similarity-based modeling. From the different features considered for mRNA, free energy, AU content, and accessibility were among the top predictors in case of mintRULS. These many features and their roles in miRNA binding have been previously discussed in the literature [14,32,65], with raising questions on their systematic integration and incorporation to predictive modeling which is still a challenge to the model developers.

### 3.4. Validation

Interacting pairs between miRNA hsa-miR-548ba and three genes which include *IFR*, *PTEN*, and *NEO1*, were classified as “Strong Target”, and showed consistency with the results in [59] (Table 2). Similarly, from the study [60], interacting pairs between miRNA hsa-miR-34a-5p and genes including *SMAD7*, *SMAD2*, *CREB1*, and *CLOCK*, were predicted as “Strong Target”, while binding of hsa-miR-34a-5p with *GRIA4* was predicted as “Weak Target”. It is interesting to notice that most predicted results are consistent with the outcomes of the experimental studies (Table 2). The interaction between these many pairs were also confirmed by performing protein level analysis in SH-SY5Y cells in the same study. Other interactions such as hsa-miR-22 with BMP-7/6, hsa-miR-146a-3p with *TRAF6* and *RIPK2*, and hsa-miR-125b with *PARP1*, p53, Beta-actin, and *CPSF6* from different studies were also verified and found consistent with the experimental outcomes (Table 2). The experimentally validated negative interactions between hsa-miR-125b and Beta-actin, and 18S RNA with gld-1:gfp mRNA were also predicted correctly as ‘Weak Targets’ (below 25th percentile) by mintRULS (Table 2).

We also checked the performance of mintRULS for predicting interactions when mutation(s) in the seed region of miRNA occur. To perform this experiment, mutation information of a few randomly selected miRNAs in human (e.g., hsa-miR-124-3p, hsa-miR-662, hsa-miR-125a-5p, etc.) and mouse (e.g., mmu-miR-342-5p, mmu-miR-690, and mmu-miR-743a-3p) along with the effects on the interactions with their target genes were downloaded from the PolymiRTS database [66]. In total, 40 pairs comprising 20 wild-type (WT) and 20 mutated (mut) miRNAs with target genes were included for this experiment. The mutation-driven changes in the interactions are described by context+ score difference (∆S), as mentioned in Table 3. Interestingly, all the WT pairs (WT miRNAs and their target genes) were predicted as “Strong Targets”, while 16 (out of 20) of their mutated counterparts were predicted as “Weak Targets”, showing good consistency with the information (∆S, representing disruption in the interaction) in the PolymiRTS database. It is noteworthy that even the other four pairs (i.e., hsa-miR-125a-5p with *ZMYM3*, hsa-miR-645 with *COL4A4*, mmu-miR-342-5p with *RASL10B*, and mmu-miR-690 with *RBBP5*) involving the mutated miRNAs were predicted as “Moderate Targets” but not as “Strong Targets”, showing that the predictions are somewhat consistent with the ∆S (Table 3). We also considered a special case study by Dash et al., 2020, where interactions of hsa-miR-124-3p with WT *PARP-1* and its mutant were observed. In this case, mintRULS performed very well by correctly classifying interactions of the miRNA with WT *PARP-1* and with four of its variants (Mut1, Mut2, Mut3, and Mut4) (Table 3).

Other than the aforementioned case specific validation, we also compared mintRULS predictions with the information in literature and databases. Table 4 listed a few of such miRNA and their target genes which are also mentioned in literature and databases, along with the mintRULS’s classifications. In most cases, the model’s classifications corroborate with the information in literature and databases, with identifying few novel interactions.

#### Supporting Predictions by Expression of miRNA and mRNA in Human and Mouse

**Figure 6:**
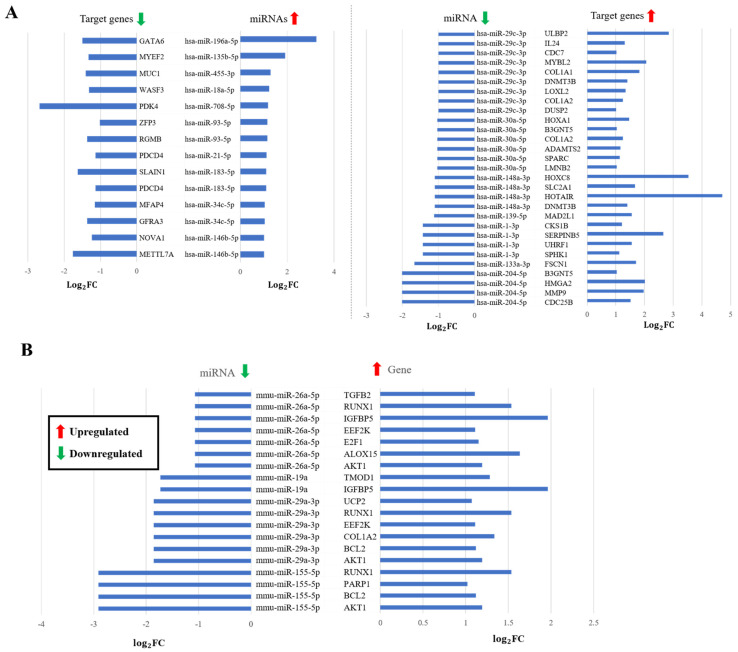
The mintRULS predicted interacting pairs in the upper quartile (>75th percentile) which have a negative correlation between miRNA and target gene expression compared in (**A**) normal vs. esophageal carcinoma human cells, and (**B**) normal vs. septic mice. The only pairs with classification “Experimental evidence” or “High prediction” in IPA analysis were considered. All the observations are significant with adj *p* value < 0.05. FC: fold change, miRNA: microRNA. For upregulation, Log_2_FC > 1, and for downregulation Log_2_FC < −1 criteria were set.

**Figure A2:**
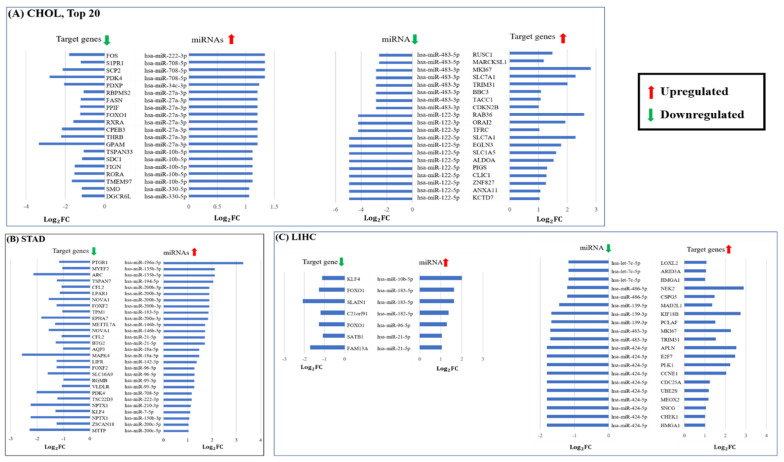
The mintRULS predicted interacting pairs in the upper quartile (>75th percentile) which have a negative correlation between miRNA and target gene expression in different gastrointestinal (GI) cancer types (**A**) cholangiocarcinoma (CHOL), (**B**) stomach adenocarcinoma (STAD), and (**C**) liver hepatocellular carcinoma (LIHC). The only pairs with classification “Experimental evidence” or “High prediction” in IPA analysis were considered. All the observations are significant with adj *p* value < 0.05. FC: Fold change, miRNA: microRNA. For upregulation, Log_2_FC > 1, and for downregulation Log_2_FC < −1 criteria were set.

Comparison between differentially expressed miRNA and genes, IPA results (“High predicted” or “Experimentally observed pairs only), showed that that most of the IPA filtered pairs were predicted either as “Strong Target” or “Moderate Target”, with only a few as “Weak Target” by our model (Table 5). In case of ESCA, 7 downregulated miRNAs were found associated with 26 upregulated target genes, while 10 upregulated miRNAs showed opposite expression correlation with 13 target genes (Figure 6A). Similarly, in LIHC, 3 upregulated miRNAs were associated with 2 downregulated genes; and conversely, 7 downregulated miRNAs showed associations with 20 upregulated target genes. We also identified 28 miRNA–gene pairs with 18 upregulated miRNAs and 24 downregulated genes in STAD. In case of CHOL, 27 downregulated miRNAs with 97 upregulated target genes, and 17 upregulated miRNAs with 58 downregulated target genes associations were identified (Figure A2, Appendix A). Not enough interacting pairs were identified in PAAD to carry forward in further analysis. Interestingly, the interacting pairs which showed experimental evidence in IPA analysis were all predicted as “Strong Target” by our method, indicating the strong predictability of the model. The detail of the interacting pairs with the FC values, IPA results, and mintRULS classifications are provided in Table S1 (Supplementary Material).

In case of mouse, analysis by GEO2R filtered in 11 differentially expressed miRNAs between normal and septic mice, while 5715 mRNA transcripts were differentially expressed. The integration of mintRULS predictions for all 11 miRNAs and the differentially expressed mRNAs identified 15 miRNA–mRNA pairs between 4 miRNAs and 10 mRNAs which also have a negative expression correlation between them (Figure 6B). The normalized predicted mintRULS score, classification, and other related information for each pair are provided in Table S2 (Supplementary Material).

## 4. Discussion

The increasing importance of miRNAs in regulating many biological processes in cells and the overall human physiology is evident from several studies. One of the major challenges in this field is the identification of functional interactions between miRNAs and target genes. The advances in sequencing technologies and the growing volume of reliable data on miRNAs and their target sites on genes have greatly facilitated studies to predict the unknown and biologically relevant interactions. Bioinformatics solutions in this realm are very diverse and inconsistent in the sense that they incorporate unique characteristics in their algorithms and provide contradictory results [77]. Several machine learning models have utilized learning features for predicting miRNA–miTS interactions but could not achieve optimal performance due to the limitations in feature selection and lack of systematic integration of multiple features.

To address some of these limitations, we employed a comprehensive list of learning features and trained them on a large experimental dataset to predict target sites with high accuracy. A special aspect of the current method includes the incorporation of pairwise similarities between various features of miRNA and miTS to improve the performance of the prediction model. The strategy for integrating pairwise correlation between miRNAs and miTS is useful for proving our hypothesis that similar miRNAs are more likely to target the same target site; and similar miTS tend to be targeted by the same miRNA. The real conditions for miRNA–miTS interactions depend on several factors such as target site accessibility [78] and complex stability [79]. mintRULS employed several of such features including binding free energy, the abundance of SSRs, and target site accessibility in the training process to develop an integrated objective scoring system. The working postulate of our method is different from those of the existing methods as evidenced by its superior prediction performance (with an AUC of 0.93) over miRDB, TargetScan, MBSTAR, RPmirDIP, and STarMir using human dataset. We attribute the performance advantage of mintRULS to its discrete feature selection and the integrated scoring function. As shown in Figure 5, the kernels built from individual features of miRNAs and miTS fairly performed with the highest AUC of 0.82, but the integrated kernel comparatively achieved higher AUC of 0.93, showing the successful integration of different sequence-derived features of miRNAs and mRNAs in a similarity-based fashion to train the model for predicting interaction pairs. The 100-fold randomization of the training dataset to train the model is extremely powerful to avoid prediction overfitting. Further, validation of predicted interacting pairs using different datasets, i.e., previous gene expression studies, literature-based findings, IPA knowledgebase with experimental and predicted interactions, and the expression data of miRNA and the target genes in four type of GI cancers (Table 5 and Table S1) showed the potential of the current model to make biologically relevant predictions. Moreover, the capability of mintRULS to predict interactions between gene and miRNAs in WT as well as mutated cases is extremely promising (Table 3).

We also demonstrated that mintRULS program can be used to predict miRNA–miTS interactions in mouse with a reasonable AUC of 0.86. The interacting miRNA-mRNA pairs that show opposite expression correlation between normal and septic mice are in support of the predictions. Negative expression correlation between miRNA and target mRNA is not a clear indication of interactions between them, but throws the high possibility, which can be confirmed by further experiments.

Overall, validation of our top predictions in human and mouse shows the robustness and superior ability of mintRULS to predict miRNA and their target site interactions. Despite obtaining high performing and reliable prediction, mintRULS have worth-noticing limitations, which mainly include lack of an experimentally validated negative dataset, and exclusion of miRNA or target abundance information. The miRNA–gene interactions are surrounded by many of the complex networks such as protein–protein interactions and gene–gene interactions, which along with the other reliable biological information could be incorporated in the future to further improve the prediction accuracy and to extend this method to predict miRNA–gene interactions in other species as well.

## 5. Conclusions

We developed a regularized least square (RLS)-based method, mintRULS, which uniquely utilizes multiple feature similarity-based metrics of miRNA and target sites to predict their interactions in human and mouse. mintRULS achieved the highest AUC of 0.93 and 0.86 in case of human and mouse, respectively. The multiple iteration and randomization strategy has helped reduce data overfitting while improving generalization and prediction performance. In comparison to other methods that include miRDB, TargetScan, MBSTAR, RPmirDIP, and STarMir, mintRULS demonstrated superior prediction ability. The model successfully utilized the existing knowledgebase as well as its unique design for pairwise incorporation of different features of miRNAs and mRNAs to predict interactions between them. Further, rigorous validation of the top predictions using multiple data sources showed outstanding capability and reliability of the model. Our method also identified new miRNA–mRNA interacting pairs such as hsa-let-7d-5p and *TIMP3*, hsa-let-7e-5p and *ZBTB7A*, and hsa-miR-106b-5p and *ATAT1*, which needs to be validated by further experimental studies.

We anticipate that the current method could be easily adopted to predict miRNA–gene interactions in other species as well to improve our knowledge of miRNA-regulated gene expression at the post-transcriptional level in different species.

## Figures and Tables

**Table 1:** Performance measurements of mintRULS by different evaluation parameter using human and mouse datasets. LOOCV: Leave-One-Out-Cross Validation, LmiTOCV: Leave-miTS-Out-Cross-Validation, ROC: Receiver Operating Characteristics, AUC: Area Under Curve, MCC: Matthews correlation coefficient.

	Accuracy	Sensitivity	Specificity	MCC	AUC (ROC Curve)
**Human dataset**					
**LOOCV**	0.908	0.847	0.909	0.67	0.931
**LmiTOCV**	0.91	0.829	0.909	0.652	0.925
**Mouse dataset**					
**LOOCV**	0.846	0.783	0.846	0.59	0.861
**LmiTOCV**	0.844	0.767	0.839	0.564	0.863

**Table 2:** Predicted miRNA-miTS interactions using mintRULS and validation using experimental data in human. Strong Target: Upper quartile (>75th percentile), Moderate Target: Middle quartile (in between 25th and 75th percentile), and Weak Target: Lower quartile (<25th percentile).

miRNA	Target Gene	Results in Reference	mintRULS		Experimental Evidence	
			Predictions (Quartile)	Classification	Cells/Tissues	Reference
hsa-miR-548ba	*LIFR*	Target	Upper	Strong Target	ovarian granulosa cells	[59]
	*PTEN*	Target	Upper	Strong Target		
	*NEO1*	Target	Upper	Strong Target		
hsa-miR-34a-5p	*CLOCK*	Target	Upper	Strong Target	SH-SY5Y cells	[60]
	*CREB1*	Target	Upper	Strong Target		
	*GRIA4*	Target	Lower	Weak Target		
	*SMAD2*	Target	Upper	Strong Target		
	*SMAD7*	Target	Upper	Strong Target		
hsa-miR-22	*BMP-7/6*	Target	Upper	Strong Target	Mouse primary kidney fibroblasts	[67]
hsa-miR-146a-3p	*TRAF6*	Target	Upper	Strong Target	Mouse Myeloid cells	[68]
	*RIPK2*	Target	Upper	Strong Target		
hsa-miR-125b	*CPSF6*	Target	Upper	Strong Target	HEK-293T	[69]
	*PARP1*	Target	Middle	Moderate Target	HEK-293T cells	[70,71]
	*p53*	Target	Upper	Strong Target		
	*Beta-actin*	Non-Target	Lower	Weak Target		
18S RNA	*gld-1:gfp*	Non-Target	Lower	Weak Target	*Caenorhabditis elegans*	[72]

**Table 3:** Validation of mintRULS predictions in case of mutations in the seed region of miRNAs or in the target gene itself. Upper quartile (>75th percentile), Moderate Target: Middle quartile (in between 25th and 75th percentile), and Weak Target: Lower quartile (<25th percentile).

miRNA	miRNA/Seed Mutation	Target Gene/Mutation	Result in Reference	mintRULS Prediction		Reference
				Quartile	Class	
hsa-miR-124-3p	UAAGGCACGCGGUGAAUGCCAA	*Parp-1* (WT)	Target	Upper	Strong Target	[73]
		Mut1: *PARP-1* (CC > GG)	No target	Lower	Weak Target	
		Mut2: *PARP-1* (TG > CA)	No target	Lower	Weak Target	
		Mut3: *PARP-1* (GC > AA)	No target	Lower	Weak Target	
		Mut4: deletion (ΔGC)	No target	Middle	Moderate Target	
cel-let-7-3p	AU[G/A]CAA	*LIN-41*	WT: Target	Upper	Strong Target	[74]
			Mutation: No Target	Lower *	Weak Target *	
hsa-miR-662	CCCAC[G/A]U	*KLLN*	Disrupted (∆S = −0.51)	Upper	Strong Target	PolymiRTS database
				Lower *	Weak Target *	
		*PATE4*	Disrupted (∆S = −0.45)	Upper	Strong Target	PolymiRTS database
				Lower *	Weak Target *	
hsa-miR-125a-5p	CCCUGA[G/U]	*ZMYM3*	Disrupted (∆S = −0.31)	Upper	Strong Target	PolymiRTS database
				Lower *	Moderate Target *	
		*PRRC1*	Disrupted (∆S = −0.45)	Upper	Strong Target	PolymiRTS database
				Lower *	Weak Target *	
		*AQPEP*	Disrupted (∆S = −0.42)	Upper	Strong Target	PolymiRTS database
				Lower *	Weak Target *	
hsa-miR-645	[C/G]UAGGCU	*COL4A4*	Disrupted (∆S = −0.38)	Upper	Strong Target	PolymiRTS database
				Middle *	Moderate Target *	
		*MAOA*	Disrupted (∆S = −0.4)	Upper	Strong Target	PolymiRTS database
				Lower *	Weak Target *	
		*IL4R*	Disrupted (∆S = −0.42)	Upper	Strong Target	PolymiRTS database
				Lower *	Weak Target *	
hsa-miR-146a-3p		*CP*	Disrupted (∆S = −0.57)	Upper	Strong Target	PolymiRTS database
				Lower *	Weak Target *	
		*ABCB1*	Disrupted (∆S = −0.35)	Upper	Strong Target	PolymiRTS database
				Lower *	Weak Target *	
mmu-miR-342-5p	[G/-]GGGUGC	*PIGU*	Disrupted (∆S = −0.46)	Upper	Strong Target	PolymiRTS database
				Lower *	Weak Target *	
		*RASL10B*	Disrupted (∆S = −0.5)	Middle	Moderate Target	PolymiRTS database
				Lower *	Weak Target *	
		*MCU*	Disrupted (∆S = −0.54)	Upper	Strong Target	PolymiRTS database
				Lower *	Weak Target *	
mmu-miR-690	AAGGCU[A/G]	*CNOT6*	Disrupted (∆S = −0.3)	Upper	Strong Target	PolymiRTS database
				Lower *	Weak Target *	
		*ELOVL4*	Disrupted (∆S = −0.35)	Upper	Strong Target	PolymiRTS database
				Lower *	Weak Target *	
		*RBBP5*	Disrupted (∆S = −0.34)	Upper	Strong Target	PolymiRTS database
				Middle *	Moderate Target *	
mmu-miR-743a-3p	AAAGAC[A/G]	*MXI1*	Disrupted (∆S = −0.33)	Upper	Strong Target	PolymiRTS database
				Lower *	Weak Target *	
		*PRRG3*	Disrupted (∆S = −0.51)	Upper	Strong Target	PolymiRTS database
				Lower *	Weak Target *	
		*MBNL3*	Disrupted (∆S = −0.43)	Upper	Strong Target	PolymiRTS database
				Lower *	Weak Target *	

Higher value of the context+ score difference (∆S) indicates an increased likelihood disruption of interactions between miRNA and target gene. * Entries for mutation in miRNAs. The values without * represents WT cases.

**Table 4:** miRNA–mRNA interactions predicted by mintRULS and supporting data in literature and databases.

miRNA	Target Gene	mintRULS		Evidence (Literature/Databases)
		Prediction Class (Quartile)	Classification	
hsa-miR-3941	*TNPO1*	Upper	Strong Target	miRDB
hsa-let-7d-5p	*BACH1*	Upper	Strong Target	TargetScan
hsa-let-7d-5p	*BCL2L1*	Upper	Strong Target	TargetScan
hsa-let-7d-5p	*NCAM1*	Upper	Strong Target	New
hsa-let-7d-5p	*TIMP3*	Upper	Strong Target	New
hsa-let-7d-5p	*IL6R*	Upper	Strong Target	TargetScan, miRDB
hsa-let-7d-5p	*CD44*	Upper	Strong Target	New
hsa-let-7d-5p	*ITGB3*	Upper	Strong Target	TargetScan, miRDB
hsa-let-7d-5p	*CCNE1*	Upper	Strong Target	miRDB
hsa-let-7d-5p	*MAP4K3*	Upper	Strong Target	TargetScan
hsa-let-7d-5p	*PTEN*	Upper	Strong Target	New
hsa-let-7e-5p	*TRIM71*	Upper	Strong Target	TargetScan, [75]
hsa-let-7e-5p	*ZBTB7A*	Upper	Strong Target	New
hsa-let-7e-5p	*KLF9*	Upper	Strong Target	TargetScan
hsa-let-7e-5p	*IGFBP5*	Upper	Strong Target	New
hsa-let-7e-5p	*ALDH5A1*	Upper	Strong Target	New
hsa-let-7e-5p	*CDK4*	Upper	Strong Target	New
hsa-let-7e-5p	*BCL2L1*	Upper	Strong Target	miRDB
hsa-let-7e-5p	*MDM4*	Upper	Strong Target	TargetScan
hsa-let-7e-5p	*TIMP3*	Upper	Strong Target	[76]
hsa-let-7e-5p	*PAPPA*	Middle	Moderate Target	TargetScan
hsa-let-7e-5p	*MYC*	Upper	Strong Target	[76]
hsa-miR-106b-5p	*NLN*	Upper	Strong Target	TargetScan
hsa-miR-106b-5p	*SLC6A4*	Upper	Strong Target	TargetScan
hsa-miR-106b-5p	*GPD2*	Upper	Strong Target	TargetScan
hsa-miR-106b-5p	*RASA1*	Upper	Strong Target	TargetScan
hsa-miR-106b-5p	*EGLN1*	Upper	Strong Target	TargetScan
hsa-miR-106b-5p	*ATAT1*	Upper	Strong Target	New
hsa-miR-106b-5p	*PAX6*	Upper	Strong Target	miRDB
hsa-miR-106b-5p	*PBX3*	Upper	Strong Target	TargetScan
hsa-miR-106b-5p	*MCL1*	Upper	Strong Target	TargetScan
hsa-miR-106b-5p	*FLT1*	Middle	Moderate Target	TargetScan miRDB
hsa-miR-106b-5p	*FXN*	Middle	Moderate Target	miRDB

**Table 5:** The summary of miRNA–target gene pairs with opposite expression correlation of associated miRNA and target genes. The only pairs which showed “Experimental evidence” or “High prediction” in IPA analysis were selected. The corresponding columns also list pairs which were predicted as “Strong Target”, “Moderate Target”, and “Weak Target”. * All the miRNA-gene pairs which showed “Experimental evidence” in IPA were predicted as “Strong Target” in mintRULS. For detailed information, Supplementary Table S1 can be referred to. IPA: Ingenuity Pathway Analysis, mintRULS predictions (Strong Target: upper quartile, >75th percentile; Moderate Target: middle quartile, >25th percentile and <75th percentile; Weak Target: lower quartile, <25th percentile), STAD: stomach adenocarcinoma, CHOL: cholangiocarcinoma, ESCA: esophageal carcinoma, LIHC: liver hepatocellular carcinoma. Upward red arrow: upregulation, downward green arrow: down regulation.

Cancer Type	Expression		IPA			mintRULS			
	miRNA	Target Gene	Exp. Observed*	High Predicted	Total	Strong-Target	Moderate-Target	Weak-Target	Total
**STAD**	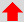	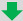	13	77	90	28	46	16	90
	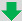	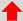	15	11	26	16	9	1	26
**CHOL**	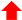	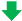	21	134	155	71	64	20	155
	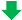	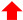	80	169	249	125	101	23	249
**ESCA**	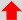	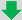	36	20	56	29	21	6	56
	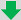	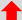	4	20	24	14	8	2	24
**LIHC**	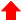	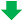	3	4	7	7	0	0	7
	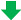	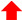	23	19	42	42	0	0	42

**Figure A3:**
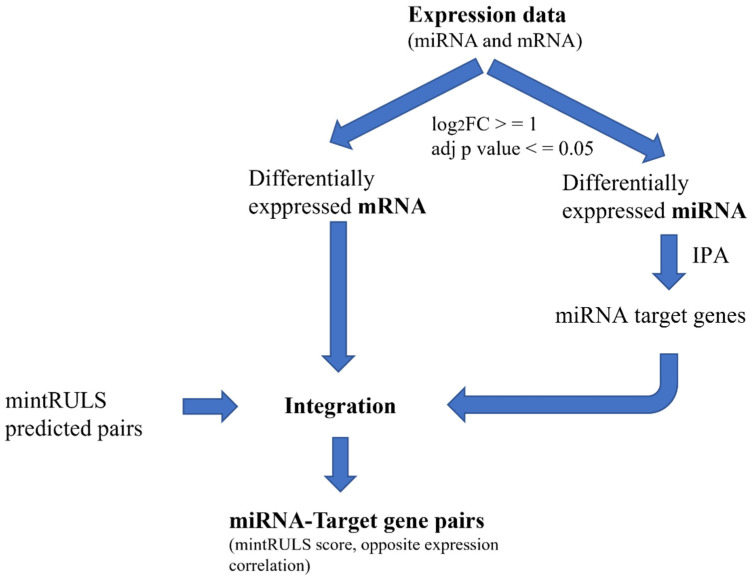
The workflow for integrating differentially expressed mRNAs and miRNA with the IPA results and mintRULS predictions.

## Data Availability

Python 3.7, PyCharm Community version 2019.3, and R 4.0.5 were used to develop scripts and run all the simulations. All the scripts and related data of mintRULS are available at https://doi.org/10.5281/zenodo.5639816.

## Supplementary Materials

The following supporting information can be downloaded at: https://www.mdpi.com/article/10.3390/genes13091528/s1, Table S1: mintRULS score, classification, and other information about validation miRNA-target interactions in GI cancer types; Table S2: mintRULS score, classification, and other information about validation miRNA-target interactions in mouse. Program code of mintRULS is freely available to the research community at: https://doi.org/10.5281/zenodo.6360587.

## Appendix A

### Appendix A.1. Methodology

#### Appendix A.1.1. miRNA/Gene Expression Analysis in Gastrointestinal (GI) Cancer

##### RNAseq Data Processing

TCGA level-3 gene expression data for pan-GI cancers (ESCA—esophageal carcinoma, STAD—stomach adenocarcinoma, COAD—colon adenocarcinoma, READ—rectum adenocarcinoma, PAAD—pancreatic adenocarcinoma, CHOL—cholangiocarcinoma, LIHC—liver hepatocellular carcinoma) containing fragments per kilobase of transcript per million mapped reads upper quartile (FPKM-UQ) data were downloaded using a R Bioconductor tool, TCGAbiolinks [80]. Differential gene expression analysis was performed using Bioconductor tool, *limma*. The genes were considered differentially expressed at a false discovery rate (FDR) < 0.05 and abs (log_2_FC ≥ 1) as a cut-off.

##### miRNAseq Data Processing

TCGA level-3 miRNASeq data for Pan-GI cancers (ESCA, STAD, READ, CHOL, PAAD, LIHC) containing reads per million (RPM) counts for each mature miRNA were downloaded from TCGA GDAC Firehose. The IDs were mapped to miRbase mature miRNA name and accession ID. We first removed all miRNA with missing expression values (in at least 25% of the samples) and also miRNA which had CPM (count per million) numbers less than one (in at least 25% of the samples). Differential miRNA expression analysis was performed using *limma* [81]. Benjamini–Hochberg (BH) adjusted *p*-value cut-off of 0.05, and an absolute log_2_ fold change (FC) of 1 was used to obtain the list of differentially expressed miRNAs. Since mature miRNA counts for normal samples were not available for READ and COAD, these cancers were not considered for further processing.

##### miRNA Target Identification Using QIAGEN Ingenuity Pathway Analysis (IPA)

Target genes of all differentially expressed miRNAs were identified using IPA Target filter (QIAGEN Inc., https://www.qiagenbioinformatics.com/products/ingenuitypathway-analysis), accessed on 20 July 2021. Further, differentially expressed miRNAs were paired to differentially expressed mRNA targets to prioritize the identified miRNA–mRNA relationship, especially the ones which have negative expression correlation.

The workflow for integrating IPA results with the mintRULS predictions are illustrated in Figure A3.

##### miRNA/Gene Expression Analysis in Control and Septic Mice

For miRNA, we considered four control samples (Accession: GSM1938976, GSM1938977, GSM1938978, and GSM1938979) and five cecal ligation and puncture (CLP)-based septic mice samples (Accession: GSM1938980, GSM1938981, GSM1938982, GSM1938983, GSM1938984) from microarray data of GSE74952 study (Affymetrix Mouse Genome 430 2.0 Array).

For mRNA, we considered four control samples (Accession: GSM1332257, GSM1332258, GSM1332259, and GSM1332260) and five CLP septic mice (at Day 1) samples (Accession: GSM1332273, GSM1332274, GSM1332275, and GSM1332276) from microarray data of GSE55238 study.

GEO2R analyzer was used to find differentially expressed miRNAs and genes. Further, a python script was developed to map mintRULS predictions and differentially expressed miRNAs/genes to identify interacting miRNA–gene pairs which have negative expression correlation.

### Appendix A.2. Calculation of Euclidean Distance Using Features

To calculate pairwise similarity between either two miRNAs or two mRNAs, the Euclidean distance (ED) was calculated by taking miRNA/mRNA’s signatures into account, as described below.

In case of miRNAs,

(A1) $$ED\left(mi_{i},\;mi_{j}\right)=\sqrt{\overset{n}{\underset{i=1}{\sum }}{\left(Fmi_{i}-Fmi_{j}\right)}^{2}}$$

$ED\left(mi_{i},\;mi_{j}\right)$ is the ED between miRNAs $mi_{i}\;\mathrm{and}\;mi_{j}$. $Fmi_{i}$ and $Fmi_{j}$ are the signatures (e.g., Free energy) of miRNAs $mi_{i}$ and $mi_{j}$, respectively.

In case of mRNAs,

(A2) $$\;ED\left(m_{i},\;m_{j}\right)=\sqrt{\overset{n}{\underset{i=1}{\sum }}{\left(Fm_{i}-Fm_{j}\right)}^{2}}$$

$ED\left(m_{i},\;m_{j}\right)$ is the ED between mRNAs $m_{i}\;\mathrm{and}\;m_{j}$. Similar to the illustration in case of miRNAs, $Fm_{i}$ and $Fm_{j}$ are the signatures of mRNAs $m_{i}$ and $m_{j}$, respectively. Here, *n* is equal to 1 for both miRNAs and mRNAs.

## References

[1] Ivey K.N., Srivastava D. (2015). microRNAs as developmental regulators. Cold Spring Harb. Perspect. Biol..

[2] Bär C., Thum T., De Gonzalo-Calvo D. (2019). Circulating miRNAs as mediators in cell-to-cell communication. Epigenomics.

[3] Harrandah A.M., Mora R.A., Chan E.K.L. (2018). Emerging microRNAs in cancer diagnosis, progression, and immune surveillance. Cancer Lett..

[4] Miranda K.C., Huynh T., Tay Y., Ang Y.S., Tam W.L., Thomson A.M., Lim B., Rigoutsos I. (2006). A Pattern-Based Method for the Identification of MicroRNA Binding Sites and Their Corresponding Heteroduplexes. Cell.

[5] Friedman R.C., Farh K.K.H., Burge C.B., Bartel D.P. (2009). Most mammalian mRNAs are conserved targets of microRNAs. Genome Res..

[6] Bartel D.P. (2009). MicroRNAs: Target Recognition and Regulatory Functions. Cell.

[7] Martin H.C., Wani S., Steptoe A.L., Krishnan K., Nones K., Nourbakhsh E., Vlassov A., Grimmond S.M., Cloonan N. (2014). Imperfect centered miRNA binding sites are common and can mediate repression of target mRNAs. Genome Biol..

[8] Helwak A., Kudla G., Dudnakova T., Tollervey D. (2013). Mapping the human miRNA interactome by CLASH reveals frequent noncanonical binding. Cell.

[9] Fabian M.R., Sonenberg N. (2012). The mechanics of miRNA-mediated gene silencing: A look under the hood of miRISC. Nat. Struct. Mol. Biol..

[10] Xu W., Lucas A.S., Wang Z., Liu Y. (2014). Identifying microRNA targets in different gene regions. BMC Bioinform..

[11] Zhang J., Zhou W., Liu Y., Liu T., Li C., Wang L. (2018). Oncogenic role of microRNA-532-5p in human colorectal cancer via targeting of the 5′UTR of RUNX3. Oncol. Lett..

[12] Kim D., Sung Y.M., Park J., Kim S., Kim J., Park J., Ha H., Bae J.Y., Kim S., Baek D. (2016). General rules for functional microRNA targeting. Nat. Genet..

[13] Liu C., Rennie W.A., Carmack C.S., Kanoria S., Cheng J., Lu J., Ding Y. (2014). Effects of genetic variations on microRNA: Target interactions. Nucleic Acids Res..

[14] Peterson S.M., Thompson J.A., Ufkin M.L., Sathyanarayana P., Liaw L., Congdon C.B. (2014). Common features of microRNA target prediction tools. Front. Genet..

[15] Kertesz M., Iovino N., Unnerstall U., Gaul U., Segal E. (2007). The role of site accessibility in microRNA target recognition. Nat. Genet..

[16] Agarwal V., Bell G.W., Nam J.W., Bartel D.P. (2015). Predicting effective microRNA target sites in mammalian mRNAs. Elife.

[17] Sticht C., De La Torre C., Parveen A., Gretz N. (2018). Mirwalk: An online resource for prediction of microrna binding sites. PLoS ONE.

[18] Bandyopadhyay S., Ghosh D., Mitra R., Zhao Z. (2015). MBSTAR: Multiple instance learning for predicting specific functional binding sites in microRNA targets. Sci. Rep..

[19] Wen M., Cong P., Zhang Z., Lu H., Li T. (2018). DeepMirTar: A deep-learning approach for predicting human miRNA targets. Bioinformatics.

[20] Pla A., Zhong X., Rayner S. (2018). miRAW: A deep learning-based approach to predict microRNA targets by analyzing whole microRNA transcripts. PLoS Comput. Biol..

[21] Kyrollos D.G., Reid B., Dick K., Green J.R. (2020). RPmirDIP: Reciprocal Perspective improves miRNA targeting prediction. Sci. Rep..

[22] Wong N., Wang X. (2015). miRDB: An online resource for microRNA target prediction and functional annotations. Nucleic Acids Res..

[23] Chen Y., Wang X. (2020). MiRDB: An online database for prediction of functional microRNA targets. Nucleic Acids Res..

[24] Kanoria S., Rennie W., Liu C., Carmack C.S., Lu J., Ding Y. (2016). STarMir tools for prediction of microRNA binding sites. Methods Mol. Biol..

[25] Vlachos I.S., Paraskevopoulou M.D., Karagkouni D., Georgakilas G., Vergoulis T., Kanellos I., Anastasopoulos I.L., Maniou S., Karathanou K., Kalfakakou D. (2015). DIANA-TarBase v7.0: Indexing more than half a million experimentally supported miRNA:mRNA interactions. Nucleic Acids Res..

[26] Chou C.H., Shrestha S., Yang C.D., Chang N.W., Lin Y.L., Liao K.W., Huang W.C., Sun T.H., Tu S.J., Lee W.H. (2018). MiRTarBase update 2018: A resource for experimentally validated microRNA-target interactions. Nucleic Acids Res..

[27] Bottini S., Pratella D., Grandjean V., Repetto E., Trabucchi M. (2017). Recent computational developments on CLIP-seq data analysis and microRNA targeting implications. Brief Bioinform..

[28] Li J., Zhang Y. (2019). Current experimental strategies for intracellular target identification of microRNA. ExRNA.

[29] Schäfer M., Ciaudo C. (2020). Prediction of the miRNA interactome—Established methods and upcoming perspectives. Comput. Struct. Biotechnol. J..

[30] Gerlach W., Giegerich R. (2006). GUUGle: A utility for fast exact matching under RNA complementary rules including G-U base pairing. Bioinformatics.

[31] John B., Enright A.J., Aravin A., Tuschl T., Sander C., Marks D.S. (2004). Human microRNA targets. PLoS Biol..

[32] Riolo G., Cantara S., Marzocchi C., Ricci C. (2021). miRNA targets: From prediction tools to experimental validation. Methods Protoc..

[33] Jiang H., Yang M., Chen X., Li M., Li Y., Wang J. (2020). MiRTMC: A miRNA Target Prediction Method Based on Matrix Completion Algorithm. IEEE J. Biomed. Health Inform..

[34] Parveen A., Mustafa S.H., Yadav P., Kumar A. (2020). Applications of Machine Learning in miRNA Discovery and Target Prediction. Curr. Genom..

[35] Plotnikova O.M., Skoblov M.Y. (2018). Efficiency of the miRNA- mRNA Interaction Prediction Programs. Mol. Biol..

[36] Zheng X., Chen L., Li X., Zhang Y., Xu S., Huang X. (2020). Prediction of miRNA targets by learning from interaction sequences. PLoS ONE.

[37] Long D., Lee R., Williams P., Chan C.Y., Ambros V., Ding Y. (2007). Potent effect of target structure on microRNA function. Nat. Struct. Mol. Biol..

[38] Fiannaca A., La Rosa M., La Paglia L., Rizzo R., Urso A. (2016). MiRNATIP: A SOM-based miRNA-target interactions predictor. BMC Bioinform..

[39] Ghoshal A., Shankar R., Bagchi S., Grama A., Chaterji S. (2015). MicroRNA target prediction using thermodynamic and sequence curves. BMC Genom..

[40] Krüger J., Rehmsmeier M. (2006). RNAhybrid: MicroRNA target prediction easy, fast and flexible. Nucleic Acids Res..

[41] Robins H., Li Y., Padgett R.W. (2005). Incorporating structure to predict microRNA targets. Proc. Natl. Acad. Sci. USA.

[42] van Laarhoven T., Marchiori E. (2013). Predicting Drug-Target Interactions for New Drug Compounds Using a Weighted Nearest Neighbor Profile. PLoS ONE.

[43] Yan C., Wang J., Lan W., Wu F.X., Pan Y. (2017). SDTRLS: Predicting Drug-Target Interactions for Complex Diseases Based on Chemical Substructures. Complexity.

[44] Yan C., Duan G., Pan Y., Wu F.X., Wang J. (2019). DDIGIP: Predicting drug-drug interactions based on Gaussian interaction profile kernels. BMC Bioinform..

[45] Yan C., Wang J., Ni P., Lan W., Wu F.X., Pan Y. (2019). DNRLMF-MDA:Predicting microRNA-Disease Associations Based on Similarities of microRNAs and Diseases. IEEE/ACM Trans. Comput. Biol. Bioinform..

[46] Chen X., Yan G.Y. (2013). Novel human lncRNA-disease association inference based on lncRNA expression profiles. Bioinformatics.

[47] Kehl T., Backes C., Kern F., Fehlmann T., Ludwig N., Meese E., Lenhof H.P., Keller A. (2017). About miRNAs, miRNA seeds, target genes and target pathways. Oncotarget.

[48] Needleman S.B., Wunsch C.D. (1970). A general method applicable to the search for similarities in the amino acid sequence of two proteins. J. Mol. Biol..

[49] Witkos T.M., Krzyzosiak W.J., Fiszer A., Koscianska E. (2018). A potential role of extended simple sequence repeats in competing endogenous RNA crosstalk. RNA Biol..

[50] Zheng Z., Reichel M., Deveson I., Wong G., Li J., Millar A.A. (2017). Target RNA Secondary Structure Is a Major Determinant of miR159 Efficacy. Plant Physiol..

[51] Liu W., Wang X. (2019). Prediction of functional microRNA targets by integrative modeling of microRNA binding and target expression data. Genome Biol..

[52] Pahikkala T., Airola A., Pietilä S., Shakyawar S., Szwajda A., Tang J., Aittokallio T. (2014). Toward more realistic drug-target interaction predictions. Brief Bioinform..

[53] Kimeldorf G., Wahba G. (1971). Some results on Tchebycheffian spline functions. J Math. Anal. Appl..

[54] Hue M., Riffle M., Vert J.P., Noble W.S. (2010). Large-scale prediction of protein-protein interactions from structures. BMC Bioinform..

[55] Rifkin R., Yeo G., Poggio T. (2003). Regularized Least-Squares Classification. Nato Sci. Ser. Sub Ser. III Comput. Syst. Sci..

[56] van Laarhoven T., Nabuurs S.B., Marchiori E. (2011). Gaussian interaction profile kernels for predicting drug-target interaction. Bioinformatics.

[57] Luo J., Xiao Q., Liang C., DIng P. (2017). Predicting MicroRNA-Disease Associations Using Kronecker Regularized Least Squares Based on Heterogeneous Omics Data. IEEE Access.

[58] Tokar T., Pastrello C., Rossos A.E.M., Abovsky M., Hauschild A.C., Tsay M., Lu R., Jurisica I. (2018). MirDIP 4.1—Integrative database of human microRNA target predictions. Nucleic Acids Res..

[59] Rooda I., Hensen K., Kaselt B., Kasvandik S., Pook M., Kurg A., Salumets A., Velthut-Meikas A. (2020). Target prediction and validation of microRNAs expressed from FSHR and aromatase genes in human ovarian granulosa cells. Sci. Rep..

[60] Kern F., Krammes L., Danz K., Diener C., Kehl T., Küchler O., Fehlmann T., Kahraman M., Rheinheimer S., Aparicio-Puerta E. (2021). Validation of human microRNA target pathways enables evaluation of target prediction tools. Nucleic Acids Res..

[61] Zhang F., Wang D. (2017). The pattern of microRNA binding site distribution. Genes.

[62] Lu L., Yu H. (2018). DR2DI: A powerful computational tool for predicting novel drug-disease associations. J. Comput. Aided Mol. Des..

[63] Singh I., Smita S., Mishra D.C., Kumar S., Singh B.K., Rai A. (2017). Abiotic stress responsive mirna-target network and related markers (SNP, SSR) in Brassica juncea. Front. Plant Sci..

[64] Patil P.G., Singh N.V., Parashuram S., Bohra A., Mundewadikar D.M., Sangnure V.R., Babu K.D., Sharma J. (2020). Genome wide identification, characterization and validation of novel miRNA-based SSR markers in pomegranate (*Punica granatum* L.). Physiol. Mol. Biol. Plants.

[65] Riffo-Campos Á.L., Riquelme I., Brebi-Mieville P. (2016). Tools for sequence-based miRNA target prediction: What to choose?. Int. J. Mol. Sci..

[66] Bhattacharya A., Ziebarth J.D., Cui Y. (2014). PolymiRTS Database 3.0: Linking polymorphisms in microRNAs and their target sites with human diseases and biological pathways. Nucleic Acids Res..

[67] Long J., Badal S.S., Wang Y., Chang B.H.J., Rodriguez A., Danesh F.R. (2013). MicroRNA-22 is a master regulator of bone morphogenetic protein-7/6 homeostasis in the kidney. J. Biol. Chem..

[68] Garo L.P., Ajay A.K., Fujiwara M., Gabriely G., Raheja R., Kuhn C., Kenyon B., Skillin N., Kadowaki-Saga R., Saxena S. (2021). MicroRNA-146a limits tumorigenic inflammation in colorectal cancer. Nat. Commun..

[69] Chaudhuri E., Dash S., Balasubramaniam M., Padron A., Holland J., Sowd G.A., Villalta F., Engelman A.N., Pandhare J., Dash C. (2020). The HIV-1 capsid-binding host factor CPSF6 is posttranscriptionally regulated by the cellular microRNA miR-125b. J. Biol. Chem..

[70] Dash S., Dash C., Pandhare J. (2021). Therapeutic significance of microRNA-mediated regulation of PARP-1 in SARS-CoV-2 infection. Non-Coding RNA.

[71] Dash S., Balasubramaniam M., Dash C., Pandhare J. (2018). Biotin-based pulldown assay to validate mRNA targets of cellular miRNAs. J. Vis. Exp..

[72] Theil K., Imami K., Rajewsky N. (2019). Identification of proteins and miRNAs that specifically bind an mRNA in vivo. Nat. Commun..

[73] Dash S., Balasubramaniam M., Martínez-Rivera F.J., Godino A., Peck E.G., Patnaik S., Suar M., Calipari E.S., Nestler E.J., Villalta F. (2020). Cocaine-regulated microRNA miR-124 controls poly (ADP-ribose) polymerase-1 expression in neuronal cells. Sci. Rep..

[74] Hunter S.E., Finnegan E.F., Zisoulis D.G., Lovci M.T., Melnik-Martinez K.V., Yeo G.W., Pasquinelli A.E. (2013). Functional Genomic Analysis of the let-7 Regulatory Network in Caenorhabditis elegans. PLoS Genet..

[75] Torres Fernández L.A., Mitschka S., Ulas T., Weise S., Dahm K., Becker M., Händler K., Beyer M., Windhausen J., Schultze J.L. (2021). The stem cell-specific protein TRIM71 inhibits maturation and activity of the pro-differentiation miRNA let-7 via two independent molecular mechanisms. RNA.

[76] Hyeon H.K., Kuwano Y., Srikantan S., Eun K.L., Martindale J.L., Gorospe M. (2009). HuR recruits let-7/RISC to repress c-Myc expression. Genes Dev..

[77] Fan X., Kurgan L. (2014). Comprehensive overview and assessment of computational prediction of microRNA targets in animals. Brief. Bioinform..

[78] Lange S.J., Maticzka D., Moḧl M., Gagnon J.N., Brown C.M., Backofen R. (2012). Global or local? Predicting secondary structure and accessibility in mRNAs. Nucleic Acids Res..

[79] Garcia D.M., Baek D., Shin C., Bell G.W., Grimson A., Bartel D.P. (2010). Weak seed-pairing stability and high target-site abundance decrease the proficiency of lsy-6 and other microRNAs. Nat. Struct. Mol. Biol..

[80] Colaprico A., Silva T.C., Olsen C., Garofano L., Cava C., Garolini D., Sabedot T.S., Malta T.M., Pagnotta S.M., Castiglioni I. (2016). TCGAbiolinks: An R/Bioconductor package for integrative analysis of TCGA data. Nucleic Acids Res..

[81] Ritchie M.E., Phipson B., Wu D., Hu Y., Law C.W., Shi W., Shi W., Smyth G.K. (2015). Limma powers differential expression analyses for RNA-sequencing and microarray studies. Nucleic Acids Res..

